# Treatment of bone pain secondary to metastases using samarium-153-EDTMP

**DOI:** 10.1590/S1516-31802004000500006

**Published:** 2004-09-01

**Authors:** Elba Cristina Sá de Camargo Etchebehere, Carlos Araújo Cunha Pereira, Mariana Cunha Lopes de Lima, Allan de Oliveira Santos, Celso Darío Ramos, Cleide Maria Silva, Edwaldo Eduardo Camargo

**Keywords:** Samarium, Pain, Metastasis, Breast cancer, Prostate cancer, Samário Dor, Metástase neoplástica, Neoplasias mamárias, Neoplasias prostáticas

## Abstract

**CONTEXT:**

More than 50% of patients with prostate, breast or lung cancer will develop painful bone metastases. The purpose of treating bone metastases is to relieve pain, reduce the use of steroids and to maintain motion.

**OBJECTIVE:**

To evaluate the use of samarium-153-EDTMP (Sm-EDTMP) for the treatment of bone pain secondary to metastases that is refractory to clinical management.

**TYPE OF STUDY:**

Retrospective.

**SETTING:**

Division of Nuclear Medicine, Universidade Estadual de Campinas (Unicamp).

**METHODS:**

Fifty-eight patients were studied (34 males) with mean age 62 years; 31 patients had prostate cancer, 20 had breast cancer, three had lung cancer, one had lung hemangioendothelioma, one had parathyroid adenocarcinoma, one had osteosarcoma and one had an unknown primary tumor. All patients had multiple bone metastases demonstrated by bone scintigraphy using TcMDP, and were treated with Sm-EDTMP. Response to treatment was graded as good (pain reduction of 50-100%), intermediate (25-49%) and poor (0-24%).

**RESULTS:**

All patients showed good uptake of Sm-EDTMP by bone metastases. Among the patients with prostate cancer, intermediate or good response to therapy occurred in 80.6% (25 patients) and poor response in 19.4% (6). Among the patients with breast cancer, 85% (17) showed intermediate or good response to therapy while 15% (3) showed poor response. All three patients with lung cancer showed poor response to treatment. The lung hemangioendothelioma and unknown primary lesion patients showed intermediate response to treatment; the osteosarcoma and parathyroid adenocarcinoma patients showed good response to treatment. No significant myelotoxicity occurred.

**DISCUSSION:**

Pain control is important for improving the quality of life of patients with advanced cancers. The mechanism by which pain is relieved with the use of radionuclides is still not yet completely understood, however, the treatment is simple and provides a low risk of mielotoxicity.

**CONCLUSION:**

Treatment with Sm-EDTMP can control the pain secondary to bone metastases effectively in most patients with breast and prostate cancer without significant side effects.

## RESUMO

**CONTEXTO:**

Mais de 50% dos pacientes com câncer de próstata, mama ou pulmão desenvolverão dor óssea secundária a metástases. O tratamento da dor óssea metastática visa minimizar a dor, reduzir o uso de opióides e manter os movimentos.

**OBJETIVO:**

Avaliar o uso de EDTMP-^153^Sm para tratamento da dor óssea secundária a metástases refratária a tratamento com opióides.

**TIPO DE ESTUDO:**

Retrospectivo.

**LOCAL:**

Divisão de Medicina Nuclear, Universi-dade Estadual de Campinas (Unicamp).

**MÉTODOS:**

58 pacientes foram estudados (34 homens), com média de idade de 62 anos. 31 pacientes com neoplasia de próstata, 20 com neoplasia de mama, três pacientes com câncer de pulmão, um com hemangioendotelioma de pulmão, um com adenocarcinoma de paratireóide, um com osteosarcoma e um paciente que apresentava um tumor primário desconhecido. Todos apresentavam múltiplas metástases ósseas à cintilografia óssea com MDP-^99m^Tc e foram tratados com EDTMP-^153^Sm. A resposta ao tratamento foi graduada em boa (redução da dor em 50 - 100%), intermediária (25-49%) e má (0-24%).

**RESULTADOS:**

Todos os pacientes apresentavam boa captação de EDTMP-^153^Sm nas metástases ósseas. Dentre os doentes com câncer de próstata, resposta intermediária ou boa ocorreu em 80.6% (25 pacientes) e má resposta em 19.4% (6). Dentre os pacientes com câncer de mama, 85% (17) apresentaram resposta intermediária ou boa à terapia enquanto 15% (3) apresentaram má resposta. Todos os três pacientes com câncer de pulmão apresentaram resposta pobre ao tratamento. Os doentes com hemangioendotelioma de pulmão e com o tumor primário desconhecido apresentaram resposta intermediária ao tratamento; os pacientes com osteossarcoma e com o adenocarcinoma de paratireóide apresentaram boa resposta. Mielotoxicidade significativa não ocorreu.

**DISCUSSÃO:**

O controle da dor é importante para melhorar a qualidade de vida do doente com câncer avançado. O mecanismo de alívio da dor com radionuclídeos ainda não foi elucidado, mas o tratamento é de simples administração e baixo risco de mielotoxidade.

**CONCLUSÃO:**

Tratamento com EDTMP-^153^Sm pode controlar a dor secundária a metástases ósseas de forma efetiva na maioria dos pacientes com câncer de próstata e câncer de mama sem efeitos colaterais significativos.

## INTRODUCTION

More than 50% of patients with prostate, breast or lung cancer will develop painful bone metastases.^1^ The prevalence of bone pain among patients with advanced malignancy is between 60 and 90%.^2^ The purpose of treating bone metastases is to relieve pain, reduce the use of steroids and maintain motion.^1^ The use of high doses of opioids causes severe side effects, including nausea, vomiting, constipation and sedation, all of which decrease the patients’ quality of life.^3^

Radiotherapy and radionuclide therapy help to diminish the opioid dose.^3^ External beam irradiation is highly effective for bone pain relief and may occasionally result in reduction of tumor mass,^1,4^ but it should not be used in multifocal metastases.^4,5^ Hemibody radiation therapy can be used in these cases, but it is associated with an unpredictable degree of toxicity, particularly when the lung and the gastrointestinal tract are radiated.^4^

Radionuclide treatment of the pain caused by bone metastases is a good option. Radionuclides are deposited in the metastatic lesion at a rate of 17:1, in comparison with the normal bone, and therefore the radiation dose to the normal bone marrow outside of the lesion is very low.^6^

The objective of this study was to evaluate the response to treatment of bone pain secondary to metastases from different tumors, using ^153^Sm-EDTMP (samarium-153-ethylenediaminetetramethylenephosphonate).

## MATERIALS AND METHODS

### Patient population

Fifty-eight patients with pain due to bone metastases and without effective control via conventional therapy were studied, after their informed consent had been obtained. Thirty-four were male and 24 were female. Their mean age was 62 years (19-85 years). Thirty-one patients had prostate cancer, twenty had breast cancer, three had lung cancer, one had lung hemangioendothelioma, one had parathyroid adenocarcinoma, one had osteosarcoma and one had an unknown primary tumor.

### Procedures for treatment with ^153^Sm-EDTMP

**Figure 1:**
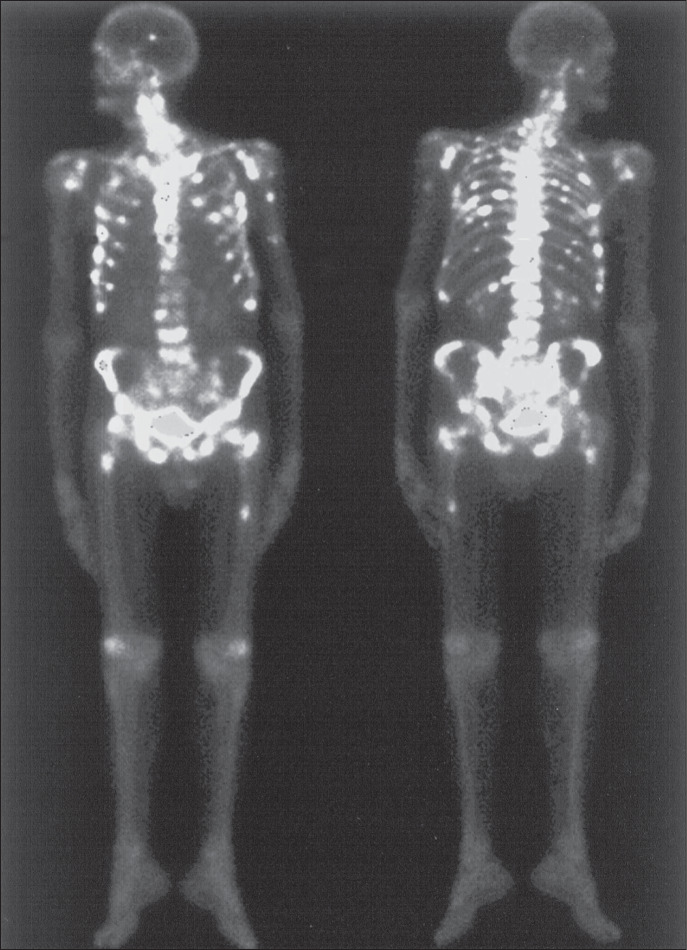
Whole body imaging using ^99m^Tc-MDP, from a patient with multiple bone metastases from breast cancer (skull, ribs, humeri, sternum, left clavicle, vertebral column, pelvis and femurs). The patient had not obtained pain relief using conventional drug therapy and was submitted to treatment using ^153^Sm-EDTMP.

The criteria utilized for including patients in this treatment protocol were:

1. Presence of pain secondary to bone metastases with no relief from conventional drug therapy and a positive bone scan using ^99m^Tc-MDP (Figure 1);
2. White blood cell count of over 2,000/μl; platelet count of over 50,000/μl; hemoglobin count of over 5.0 g/dl.

On the day of the treatment with ^153^SmEDTMP, patients were required to quantify their pain on a scale from 0 to 10 (score 0 = no pain; score 10 = maximum pain).^3^ The objective criteria for pain quantification that the patients were asked to use as guides for pain scoring were:

1. Whether the patient was still able to walk;
2. Whether the patient was waking up because of the pain;
3. Whether the patient needed help for eating, walking and personal hygiene.

The injection protocol utilized was as follows. Patients received an intravenous injection of 37-59.2 MBq/kg (1.0-1.6 mCi/kg) of ^153^Sm-EDTMP. Forty-three patients (74.2%) received 37 MBq/kg, one (1.7%) received 40.7 MBq/kg, four (6.9%) received 44.4 MBq/kg, four (6.9%) received 48.1 MBq/kg, one (1.7%) received 55.5 MBq/kg, three (5.2%) received 59.2 MBq/kg and two (3.4%) received 59.2 MBq/kg.

Whole body imaging in the anterior and posterior positions were obtained four hours after dose administration and the patients remained in the nuclear medicine laboratory during this period. Three nuclear medicine physicians read the images.

Blood tests were performed before treatment with ^153^Sm-EDTMP and after 3, 4 and 6 weeks had passed. The pain score was monitored on a monthly basis. The response to treatment was considered to be good when the pain score decreased by 50-100%. It was considered to be intermediate when the pain score decreased by 25-49% and poor when the pain score decreased by 0-24%.

### Statistical analyses

The chi-squared test and Fisher exact test were used to verify associations or compare proportions. The chi-squared test was used for comparison of responses to therapy versus age and leukocyte counts versus dose. The Fisher exact test was used for comparison of response to therapy versus tumor type or dose, and platelet counts versus dose.

For comparisons of continuous variables, the Mann-Whitney test was used for pairs of groups, and the Kruskal-Wallis test for three or more groups (response to treatment compared with age, dose and different types of tumor).

When two different time intervals were compared in the same sample, such as the comparison between the initial pain score and the pain score after six months, in relation to tumor type, the Wilcoxon test was applied.

To verify linear associations between pairs of continuous variables, such as the comparison between doses and blood counts, regardless of the tumor type, the Spearman linear correlation coefficient was used. The level of significance used was 5%.

## RESULTS

All 58 patients showed good uptake of ^153^Sm-EDTMP by bone metastases (Figure 2). Among them, 32 (55.2%) were good responders to treatment with ^153^Sm-EDTMP, 13 (22.4%) were intermediate responders and 13 (22.4%) were poor responders. No significant difference was noted between patient outcome and tumor type, when the tumors were grouped as prostate, breast and other tumors (p = 0.0846; Fisher exact test) (Table 1).

**Table 1:** Patient outcome in a protocol of bone pain treatment, according to tumor type; dispersion values for white blood cells and platelet counts per mm^3^ at the 4^th^ week after treatment using 153Sm-EDTMP; initial pain score and pain score after six months, according to tumor type

Tumor type	Outcome				Serum levels			Pain score		
	Good (%)	Intermediate (%)	Poor (%)	Total (%)	Variable	n	Mean ± SD	Timing	Mean ± SD	p value*
**Prostate**	16	9	6	31	WBC	31	4,951 ± 2,115	Initial	7.4 ± 2.0	< 0.0001
	(28.0)	(15.8)	(10.5)	(54.4)	Platelets	31	143,632 ± 76,809	Final	3.5 ± 2.5	
**Breast**	14	3	3	20	WBC	20	4,010 ± 2,075	Initial	7.1 ± 2.3	< 0.0001
	(24.6)	(5.3)	(5.3)	(35.1)	Platelets	20	13,1310 ± 92,843	Final	3.0 ± 2.9	
**Other**	2	0	4	6	WBC	6	5,395 ± 2,630	Initial	8.0 ± 2.1	0.1250
	(3.5)	(0)†	(7.0)	(10.5)	Platelets	6	137,000 ± 60,956	Final	6.2 ± 3.4	
**Total** ‡	32	12	13	57	WBC	58	4,620 ± 2,186	Initial	7.4 ± 2.1	-----
	(56.1)	(21.1)	(22.8)	(100)	Platelets	58	139,490 ± 79,886	Final	3.6 ± 2.8	

*SD = standard deviation; WBC = white blood cells.*

* *Wilcoxon test for related samples;*

† *Frequency of missing data = 1 (refers to patient #58, whose primary tumor was unknown): percentages are calculated in relation to a sample of 57 patients;*

‡ *p = 0.0846; Fisher exact test.*

The 32 good responders remained free from pain for an average of 5.75 months, and 22 patients (68.75%) remained without pain for at least six months.

Sixteen of the 31 patients with prostate cancer were good responders, nine were intermediate responders and six were poor responders (Table 1). Fourteen of the 20 patients with breast cancer were good responders, three were intermediate responders and three were poor responders (Table 1). All three patients with lung cancer showed poor response to treatment with ^153^Sm-EDTMP.

The treatment response was not influenced by gender (p = 0.5923; chi-squared test) or age (p = 0.4941; Kruskal-Wallis test). However, in the subgroup of older patients with prostate cancer, a better response to treatment was noted (p = 0.0276; Kruskal-Wallis test). There was a significant decrease in the pain score after treatment, in comparison with the pre-treatment score, among patients with breast and prostate cancer (p < 0.0001; Wilcoxon test) (Table 1).

No significant difference was observed between the administered dose and the treatment response, for patients with prostate cancer (p = 0.8754; Fisher exact test), breast cancer (p = 0.7887; Fisher exact test) and other tumor types (p = 0.7893; Fisher exact test).

A reduction in platelet and white blood cell counts was noted. At a time of three to four weeks after treatment, twenty patients (34%) had platelet counts of less than 100,000/mm^3^ and four patients (6.9%) had less than 50,000/mm^3^, although no bleeding occurred. All count levels had returned to normal by six weeks after the treatment (Table 1).

There was no significant correlation between the platelet count variation and the administered dose (p = 0.8824; ρ = 0.01986; Spearman linear correlation coefficient). Nor was there between the white blood cell count variation and the administered dose (p = 0.9743; ρ = 0.00432; Spearman linear correlation coefficient).

## DISCUSSION

Approximately 60% of patients with metastatic cancer suffer from bone pain, which limits the individual's autonomy and social life. Control of such pain is therefore important for improving patients’ quality of life.^7^ External beam radiotherapy can reach bone pain relief rates of 80-85%, but this method cannot be used to treat multiple lesions. Medical therapy may also not be effective in treating patients with disseminated bone metastases. Many of these treatments are limited in their efficacy or duration and have significant side effects that seriously limit the patients’ quality of life.^8^

Therapy using radionuclides deposits high doses of radiation in bone lesions, in comparison with the deposition in normal bone, with ratios ranging from 4:1 to 17:1, and such therapy is therefore of great use in the treatment of disseminated metastatic bone disease.^1^

The kinetics and biodistribution of ^153^SmEDTMP and ^99m^Tc-MDP are very similar.^9^ ^153^Sm-EDTMP has high affinity for skeletal tissue and concentrates in areas with high bone metabolism. Within two to three hours after injection, 50% to 66% of the administered dose concentrates in bone, and 33% to 50% is excreted by the kidneys.^1^ Less than 2% of the administered dose is localized in non-osseous tissues, and this is mainly in the liver.^1,10^

The use of radionuclides for treatment of bone metastases has the aims of decreasing pain and the use of analgesics, and improving the quality of life.^11,12^ In a study in which twenty patients with osteoblastic metastases were treated with ^153^Sm-EDTMP, the amount of analgesic therapy was reduced in 79% of the patients.

The mechanism responsible for pain relief has not been entirely elucidated. The reduction of the intra-medullary pressure does not account for the rapid pain relief that may occur a few days after the radionuclide administration, since the absorbed radiation dose delivered has not yet destroyed a sufficient quantity of tumor cells.^2^ One possible explanation would be that tumor necrosis induced by radiation would result in the death of cells that participate in the inflammatory and immuno-logical processes, consequently reducing the release of bradykinins, tumor necrosis factor, prostaglandins and interleukins, substances that are known to increase pain.^2,3^

The criteria used to measure the pain experienced by the patients in the present study came from the perspective of other clinicians with wide experience in the subject. “The assessment of bone pain in patients with cancer involves careful validation of quality, intensity and site of pain. It is suggested that some simple pain measurement be used to consistently rate and document the intensity of the patient's pain and the degree of pain relief with therapeutic interventions. Several such scales exists, as well as an even simpler ‘0-10’ numerical rating of pain relief. It is critical that the patients, not the physician or nurse, be allowed to measure their pain; numerous studies have documented how often second party observers underrate the intensity of pain and overrate the degree of pain relief experienced by patients.”^3^

The main side effect from treatment is myelotoxicity.^14^ In the present study, platelet counts of less than 50,000/mm^3^ occurred in a minority of patients (7%), which is in agreement with the current literature.^1,4^

Among 107 patients with painful bone metastases who were submitted to a dose-controlled treatment study using ^153^Sm-EDTMP, 13% developed myelotoxicity, with platelet counts of less than 50,000/mm^3^.^4^ Another trial, in which 34 patients with painful multifocal skeletal metastases were treated using single and repeated doses of ^153^Sm-EDTMP, found similar results, with platelet counts of less than 50,000/mm^3^ in 13%.^10^ No bleeding episodes were reported in these two studies.

Eary et al.^9^ administered different doses and noted that the highest tolerable dose was 92.5 MBq/kg. Although myelotoxicity could not be predicted, it was easily controlled with platelet transfusion.^9^ Resche et al.^4^ observed pain relief in 55% of patients that received 18.5 MBq/kg of ^153^Sm-EDTMP, while 70% of patients that received 37 MBq/kg had pain relief. These authors also observed a higher survival rate among those patients with breast cancer that received 37 MBq/kg. The dose can be repeated, if clinically indicated.^4^ Menda et al.^15^ treated one prostate cancer patient more than once, obtaining good pain control with little reduction in platelet and white blood cell counts.

In the present study, among the patients that received 37 MBq/kg (74%), 77% had pain relief, although a better response was not seen with doses above 37 MBq/kg. The administered dose had no direct effect on patient outcome. In 78.9% of the patients, there was good or intermediate treatment response, which is in agreement with the current literature.^13,14,16,17^ In another study, a single dose of ^153^Sm-EDTMP in the palliative treatment of 72 patients with painful skeletal metastases provided palliation in 83.8% of the patients.^14^

Among a group of 54 patients treated using ^153^Sm-EDTMP, excellent results were seen in 66%, with complete pain control.^17^ Turner et al.^10^ studied 28 patients with disseminated skeletal metastases who were submitted to therapy using ^153^Sm-EDTMP, and noted pain relief in 79%. The response duration ranged from 4 to 35 weeks, similar to the results in the present study.

Systemic treatment using radionuclides is simple to administer, with few side effects. It improves patient mobility, reduces patients’ dependence on steroidal and non-steroidal analgesics and improves patients’ quality of life and survival.^12^

## CONCLUSION

Treatment with ^153^Sm-EDTMP can control the pain secondary to bone metastases^18^ effectively in most patients with breast and prostate cancer without significant side effects.
